# Disruption of Epistemic Trust in Borderline Personality Disorder: A Possible Adaptation to Avoid Making Costly Mistakes

**DOI:** 10.1002/pmh.70006

**Published:** 2025-01-22

**Authors:** Yağızcan Kurt

**Affiliations:** ^1^ Department of Clinical, Educational and Health Psychology University College London London UK; ^2^ Anna Freud London UK; ^3^ Department of Psychology Istanbul Medeniyet University Istanbul Turkey

**Keywords:** borderline personality disorder, epistemic credulity, epistemic mistrust, error management theory, signal detection theory

## Abstract

This paper applies *error management theory* (EMT) (Haselton and Buss 2000) to explore how disruptions in *epistemic trust*—trust in communicated information—can be understood as adaptive responses to early adversity in individuals with borderline personality disorder (BPD). I propose that *epistemic mistrust* (EM) and *epistemic credulity* (EC), characterized by inappropriate trust patterns, arise from the differential costs of trusting unreliable versus mistrusting reliable information. Although these biases may seem maladaptive, they function as evolutionary survival mechanisms in response to harsh environments. Signal detection analysis can provide empirical evidence for these trust biases by assessing how individuals with BPD make trust‐related decisions. Clinically, understanding these biases as evolutionary adaptations helps reduce stigma and informs evolutionary‐informed interventions to recalibrate trust responses and improve interpersonal relationships. This approach highlights the significance of integrating evolutionary perspectives in treating trust disturbances in BPD.

## 1 Introduction

Recent research in the field of developmental psychopathology highlights the need to consider the developmental antecedents and trajectories of personality disorders in order to understand these disorders (Altschuler and Krueger 2021; Cicchetti 2014; De Fruyt and De Clercq 2014). Borderline personality disorder (BPD) is among the most frequently investigated personality disorders in the context of developmental psychopathology (Chanen and Kaess 2012; Goodman et al. 2013; Hecht et al. 2014). In addition to empirical investigations, aetiological accounts of BPD also emphasize early adverse experiences coupled with genetic vulnerabilities in the development of the disorder (Fonagy and Luyten 2009; Kellogg and Young 2006; Kernberg 1986; Linehan 1993).

The mentalizing theory, a theory that explains the problems in BPD, has recently been extended to include *epistemic trust*, that is, the capacity to find the information conveyed by others reliable, self‐relevant and transferable to other contexts (Fonagy and Allison 2014). This approach underlines the role of such trust in enabling salutogenesis, the process of leveraging positive elements in one's environment to overcome adversity through social learning (Campbell et al. 2021; Luyten, Campbell, Allison, and Fonagy 2020). Conversely, *epistemic mistrust* (EM)—a lack of trust in communicated knowledge—or *epistemic credulity* (EC)—an excessive and inappropriate trust in communicated knowledge—may be linked to core aspects of psychopathology, such as rigidity and instability, respectively (Fonagy, Luyten, and Allison 2015; Luyten and Fonagy 2022).

Although EM and EC have been described as adaptive reactions to challenging early environments (Luyten, Campbell, and Fonagy 2020), this claim needs to be further investigated from an evolutionary perspective, as the term *adaptive* in this context has a particular connotation pertaining to the field of evolutionary sciences. Previous theory and research have mainly focused on the adaptive nature of EM and EC using an analysis at the individual and developmental level; thus, a theoretical evolutionary account may be of importance to establish whether these stances indeed serve adaptive functions in the context of dynamic evolutionary processes.

The theory of epistemic trust in BPD appears to lack a comprehensive theoretical explanation for adaptiveness within an evolutionary science framework, leaving the underlying mechanisms insufficiently addressed. To enhance its claims, the theory could benefit from proposing a clear evolutionary mechanism to explain why these processes might be considered adaptive. This theoretical gap seems further accentuated by the limited empirical evidence currently available to support its assertions. An evolutionary explanation has the potential to address these conceptual limitations and provide a framework that is both empirically testable and scientifically grounded.

Building on this background, this paper aims to examine the adaptiveness of EM and EC through the lens of *error management theory* (EMT) (Haselton and Buss 2000). This theory argues that human judgment has evolved to include systematic biases to minimize costs when false‐positive and false‐negative errors carry unequal risks. Using this theory, I suggest that both EM and EC can be understood as adaptive biases shaped by evolutionary pressures.

Adopting an evolutionary framework for understanding the adaptiveness of disrupted epistemic trust could help address the gap in the current theory, which posits such disruption as adaptive yet lacks a clear mechanistic explanation. Additionally, building on EMT—which is grounded in signal detection theory (SDT) (Green and Swets 1966; Swets, Dawes, and Monahan 2000)—provides a systematic framework for examining how individuals evaluate the trustworthiness of communicated knowledge and determine thresholds at which they choose to trust or mistrust it. By applying SDT in this context, researchers may be able to develop testable predictions about EM and EC. Through this approach, an evolutionary framework could not only enrich the theory of epistemic trust but also offer a more structured path for empirical inquiry, potentially leading to a stronger theoretical and evidence‐based foundation.

Integrating the evolutionary psychopathology perspective into the developmental psychopathology context, this article attempts to contribute to understanding how early deprivation and abuse can lead to apparently maladaptive dynamics of trust in individuals with BPD. I first examine the role of epistemic trust in BPD, then examine how EMT may explain impairments in epistemic trust.

## 2 The General Factor of Psychopathology and BPD

In recent years, in light of the limitations of categorical approaches to mental disorders, there has been increasing argument that a dimensional classification of mental disorders may be more accurate. The argument highlights research showing that psychopathology exists on a continuum, categorical diagnoses often have low reliability, diagnostic processes are heterogeneous—allowing individuals with different symptoms to receive the same diagnosis—and comorbidity rates among mental disorders are high (Kotov et al. 2017).

In line with this dimensional approach, two higher‐order factors from mental disorder symptoms were identified: internalizing and externalizing (Achenbach 1966). However, the strong association between these factors prompted further investigation into a broader factor of psychopathology (Smith et al. 2020). Caspi et al. (2014) found that psychopathology is better explained by one general factor than by three higher‐order factors (internalizing, externalizing and thought dysfunction). Several studies have replicated the existence of this general factor of psychopathology (Castellanos‐Ryan et al. 2016; Lahey et al. 2012; Sallis et al. 2019). Caspi et al. (2014) coined the term *p factor* to describe this single underlying dimension, which reflects an individual's likelihood of developing various forms of psychopathology. A higher p factor score is associated with greater impairment, stronger familial psychopathology tendencies, more challenging developmental histories and more severe early‐life brain function deficits (Caspi et al. 2014).

A similar statistical construct, termed *g‐PD*, has been identified for personality pathology (Oltmanns et al. 2018; Sharp et al. 2015; Wright et al. 2016). Sharp et al. (2015) found that the diagnostic criteria for BPD loaded solely onto the general factor, whereas other personality disorders loaded onto both the general and a specific factor, or primarily onto a specific factor. This led the authors to suggest that BPD might potentially define *g‐PD*. In other words, the criteria for BPD may reflect the fundamental elements of personality pathology (Fonagy et al. 2017).

Building on Sharp et al. (2015), it is thought that the features of BPD indicate the persistence of psychopathology (Luyten and Fonagy 2022). To account for this persistence, Fonagy et al. (2022) suggest that many forms of psychopathology may arise from social communication difficulties, specifically a lack of trust in others as sources of knowledge about the self and others. They suggest that disruption in epistemic trust, and the resulting impairment in social learning, could be a common feature across various psychopathologies. Consequently, the *p factor* may act as a proxy for EM, which leads to diminished social learning capacity and increased vulnerability to persistent psychopathology.

## 3 Epistemic Trust and the Intergenerational Transmission of Cultural Knowledge

Epistemic trust is defined as the capacity to regard new information communicated through interpersonal interactions as reliable, personally meaningful and applicable across contexts (Fonagy and Allison 2014). Evolutionarily, this ability enables the acquisition of culturally relevant knowledge from more knowledgeable sources (Csibra and Gergely 2011). Many objects essential for survival are cognitively opaque, meaning their use cannot be inferred from appearance alone. A common example is a hammer, which a child would struggle to understand without guidance from someone more experienced (Csibra and Gergely 2009; Fonagy, Luyten, and Allison 2015). The cognitive opacity of such cultural artefacts creates a challenge for intergenerational knowledge transmission. To solve this, one can either attempt to independently understand the tools—often a laborious process—or rely on the knowledge of others (Csibra and Gergely 2011; Fonagy et al. 2017). Trust in the knowledge communicated by others facilitates faster, more efficient cultural transmission, allowing learners to encode information generically rather than contextually (Fonagy et al. 2017). Csibra and Gergely (2009) suggest that human communication evolved to support the rapid transfer of cognitively opaque knowledge across generations.

Although epistemic trust is key to acquiring survival‐critical information, excessive trust could be disadvantageous in uncertain conditions. To guard against misinformation, humans have evolved epistemic vigilance, a mechanism to rationally assess the trustworthiness of communicated knowledge (Sperber et al. 2010). Studies show that even children evaluate information for accuracy before accepting it (Brosseau‐Liard 2017; Koenig and Harris 2005). Neurochemicals like oxytocin and dopamine, associated with trust, need to be activated to override the default state of epistemic vigilance (Bartz et al. 2011). This suggests that cautiousness about the reliability of information was adaptive in our ancestral environment, where the cost of being misled could be high.

Epistemic trust, by facilitating the distinction between beneficial and harmful information, is crucial for establishing and maintaining culture. Although epistemic vigilance serves as a default, trust is essential for survival through the transmission of knowledge that sustains human culture (Fonagy et al. 2022). Recognizing this role is critical for understanding how culture is perpetuated through trust in interpersonally communicated information.

Transmitting cultural knowledge requires the ability to *mentalize*—the capacity to understand one's own and others' behaviours in terms of mental states (Fonagy and Allison 2014). Additionally, *joint attention*—focusing on an object alongside another person from different perspectives—is essential (Tomasello 1999). Though evidence of mentalizing exists in nonhuman primates and other animals (Rosati and Hare 2009), humans have a unique capacity for joint attention (Tomasello 2020). This is characterized by shared intentionality, where individuals recognize others as intentional agents while pursuing a common goal (Gallotti and Frith 2013). This is described as the *we‐mode* (Tomasello 2020) or relational mentalizing (Asen and Fonagy 2012, 2017), which fosters cooperation, the recognition of subjectivity and the transmission of cognitively opaque knowledge. The we‐mode, unlike the self‐serving *I‐mode*, opens the pathway for cultural transmission, enabling the continuity of culture across generations (Fonagy et al. 2022).

In line with the emphasis on coordinated minds and cooperation, Csibra and Gergely's (2009) *theory of natural pedagogy* provides a framework to explain how epistemic trust develops despite the default state of epistemic vigilance. They introduced the concept of *ostensive cues*—communicative signals such as sustained eye contact, direct address, widened eyes, forward body posture, pointing gestures and raised eyebrows (Allen 2022). These cues indicate the communicator's intent and enable recipients to encode the information for future use. Human infants demonstrate a species‐specific sensitivity to these cues (Csibra and Gergely 2009). When ostensive cues are used, the recipient is more likely to trust the communicated information, as they experience a recognition of being perceived as an intentional agent (Allen 2022; Benjamin 2018). This leads to the *pedagogical stance*, allowing for the acquisition of culturally relevant, meaningful and generalizable knowledge from others (Csibra and Gergely 2009, 2011). Thus, ostensive cues help overcome the natural barrier of epistemic vigilance and facilitate the transfer of cultural knowledge across generations.

Several studies have empirically supported the natural pedagogy theory, demonstrating the role of ostensive cues in various aspects of learning. For instance, Senju and Csibra (2008) found that 6‐month‐old infants were more likely to follow an experimenter's gaze in response to ostensive cues than to non‐social cues. Other studies have shown that infants expect referential and generalizable information after ostensive cues (Csibra and Volein 2008; Yoon, Johnson, and Csibra 2008), with effects on word learning (Axelsson, Churchley, and Horst 2012), object property processing (Marno, Davelaar, and Csibra 2014), information transfer (Vredenburgh, Kushnir, and Casasola 2015) and imitation (Buchsbaum et al. 2011).

However, recent research has questioned the validity of findings supporting natural pedagogy, suggesting that the effects of ostension could be attributed to domain‐general attentional processes rather than a domain‐specific evolutionary mechanism (Gredebäck, Astor, and Fawcett 2018; Szufnarowska et al. 2014). However, one study found that although 9‐month‐old infants responded similarly to both ostensive and attentional cues in terms of gaze following, only ostensive cues influenced their referential object learning (Okumura et al. 2020). This may indicate that ostensive cues have a unique role in social learning, as predicted by natural pedagogy, though further research is needed to clarify their distinct effects beyond attentional processes.

## 4 Epistemic Trust in a Developmental Psychopathology Framework

Fonagy, Luyten, and Allison (2015) explored epistemic trust within a developmental psychopathology context, suggesting that reduced epistemic trust may explain the rigid behavioural patterns often seen in mental disorders. A diminished capacity to trust knowledge from others can disrupt social learning, hindering the individual's ability to benefit from meaningful, applicable and generalizable information. This disruption may impair adaptation to a changing environment, thereby decreasing resilience—an essential feature of psychological disorders (Luyten and Fonagy 2022).

Epistemic trust is thought to be shaped by early attachment relationships (Fonagy, Luyten, and Allison 2015). Secure attachment is associated with reduced epistemic vigilance, as factors like caregiver sensitivity (Ainsworth et al. 1978) and parental reflective functioning (Slade 2005) not only contribute to secure attachment but also promote the development of epistemic trust. Sensitive and contingent interactions between caregiver and infant signal that the infant is perceived as an intentional agent, fostering trust in communicated knowledge and conferring the cognitive benefits linked to secure attachment (Fonagy et al. 2022).

In contexts of early abuse or deprivation, it may be functional for infants to mistrust communicated knowledge due to caregivers' hostile intentions (Fonagy et al. 2017). Infants in such environments may not receive adequate or appropriate ostensive signals to develop trust in communicated knowledge, or these signals may be misused to convey harmful information. Mistrusting communicated knowledge may thus be a protective adaptation in hostile environments (Campbell et al. 2021). However, chronic mistrust can lead to selective EC, where individuals lack the ability to apply rational doubt to the reliability of information, making them vulnerable to exploitation.

Consistent with this developmental psychopathology model, research shows that the quality of the relationship between a child and a communicator significantly affects the child's ability to learn from and apply communicated information (Corriveau et al. 2009; Lane and Harris 2015; Mascaro and Sperber 2009). Although initial evidence supports the importance of these relationships for epistemic trust, further research is needed to test this developmental psychopathology approach.

## 5 Clinical Presentation of Disruption of Epistemic Trust and Psychotherapy

EM has been shown to have a chronic impact on individuals, impairing their ability to engage in social learning and update their understanding of the self, others and the environment (Fonagy et al. 2017). This disruption can lead to suboptimal performance in social situations requiring such knowledge, placing individuals at a disadvantage.

To comprehend both the world and the self, we rely on the knowledge of others. An example of this perspective is expressed by Proust (2013), who states, ‘our social personality is a creation of the minds of others’ (p. 21). This view suggests that our self‐understanding is not derived solely from internal experiences but is shaped and updated through complex social interactions.

A reciprocal relationship between one's self‐image and how others perceive them is crucial for social learning (Fonagy et al. 2022). When others' representation of the individual matches the individual's self‐image, a co‐representation is formed, leading to the establishment of a we‐mode that facilitates efficient knowledge transmission. This shared understanding fosters openness to learning from others.

Empirical studies (Chiesa and Fonagy 2014; Ensink et al. 2017; Levy, Goldstein, and Feldman 2019) have linked compromised mentalizing capacity, often resulting from early abuse or deprivation, to distorted understandings of social reality. For example, Chiesa and Fonagy (2014) found that impaired mentalizing mediates the relationship between child maltreatment and personality disorder symptoms. Such distortions may arise from avoiding mentalizing as a protective strategy against perceived hostile intentions (Fonagy et al. 2022), leading to an incompatibility between an individual's personal narrative and social reality.

Difficulties with mentalizing can distort an individual's self‐image and perception of how others view them. Even when others accurately represent their self‐image, individuals may feel misunderstood if this representation does not align with their internal reality (Fonagy et al. 2022). This lack of alignment—absence of we‐mode—can inhibit trust in social information. Moreover, reduced capacity to mentalize others' intentions can prevent individuals from recognizing cues of care and acceptance, whereas an unintegrated self‐view may lead to EC, leaving them vulnerable to misinformation.

EM and EC are distinct yet related constructs (Campbell et al. 2021). Fonagy, Luyten, and Allison (2015) suggest that a mismatch between self‐representation and how others perceive the individual may result in isolation, driving epistemic hunger and leading to EC. This oscillation between mistrust and credulity is frequently observed in individuals with personality pathology (Kamphuis and Finn 2019), indicating that epistemic trust, mistrust and credulity are three related but distinct factors rather than points on a spectrum.

Fonagy, Luyten, and Allison (2015) developed a three‐tiered model to explain psychotherapy's effectiveness for BPD. The model includes three communication systems: epistemic match, improved mentalizing and re‐emergence of social learning outside therapy. Initially, through epistemic match, therapy provides patients with a framework for understanding their mental states. The therapist reduces epistemic vigilance by mentalizing the patient's experiences with sensitivity, enabling the patient to become open to learning. This leads to improved mentalizing, allowing for a more accurate understanding of the self and others. Finally, for therapeutic gains to generalize to the patient's social life, they must develop epistemic trust in their external environment. Without this trust, therapeutic progress may not translate beyond therapy.

## 6 EMT and the Disruption of Epistemic Trust

In light of the theoretical background on the association between impaired epistemic trust and BPD, there are arguments that EM is an adaptive mechanism resulting from childhood maltreatment. For the use of the word *adaptive* to be justified, the applicability of this claim in terms of evolutionary sciences should be analysed. Moreover, theoretical arguments regarding the origins and adaptive aspects of EC are limited. An evolutionary theoretical account is necessary for both EM and EC. In this respect, EMT (Haselton and Buss 2000) may offer an evolutionary explanation that captures the adaptive nature of these trust dynamics. To better understand EMT, it is essential to first provide background information on SDT (Green and Swets 1966; Swets, Dawes, and Monahan 2000).

SDT focuses on decision‐making in uncertain environments, where four possible outcomes exist: true positive (correct detection of a signal), false positive (detecting a signal when none is present), true negative (correctly rejecting the absence of a signal) and false negative (failing to detect a signal when it is present). In such scenarios, individuals must process noise (random stimuli that interfere with the signal) to accurately detect the target signal. Decision outcomes are influenced by two key components: response bias (the tendency to detect or not detect a signal) and sensitivity (the ability to accurately distinguish between signal and noise) (Stanislaw and Todorov 1999). Decreased sensitivity is typically linked to problems in information processing (Frith 1979; Martinez et al. 2021), whereas response biases are shaped by perceived benefits and costs associated with each possible response (Haselton and Buss 2000; Haselton and Nettle 2006).

To better understand disruptions in epistemic trust, distinguishing between sensitivity issues and response bias may be helpful. I argue that response bias is more relevant in this context, as sensitivity typically relates to broader information‐processing deficits. Specifically, EM can be conceptualized as a response bias towards perceiving information as untrustworthy, whereas EC can be seen as a response bias towards perceiving information as trustworthy.

Response biases in individuals with disrupted epistemic trust can be understood through EMT (Haselton and Buss 2000; Haselton and Nettle 2006). This theory posits that human perception and cognition have evolved to exhibit systematic biases when the costs of making false‐positive and false‐negative errors are imbalanced.

Haselton and Nettle (2006) argue that the relative costs of false‐positive and false‐negative errors vary depending on the context. For instance, in hypothesis testing, false positives are costlier than false negatives, leading to a decision‐making bias that favours avoiding false positives. Conversely, in hazard detection, false‐negative errors may be costlier than false‐positive ones. This principle, known as the *smoke detection principle* (Nesse 2001), explains why systems like smoke detectors are biased to produce more false alarms (false positives) to minimize the risk of missing a genuine threat (false negatives). Applying this principle to epistemic trust, individuals may develop biases to either overdetect or underdetect trustworthiness based on the perceived risks of being misled.

When there is an imbalance between the costs of false‐positive and false‐negative errors, human‐designed systems tend to exhibit a bias towards making the less costly error (Haselton and Nettle 2006). Although this can lead to an increase in total errors, the overall cost of those errors is reduced (D. M. Green and Swets 1966; Haselton and Nettle 2006). Haselton and Buss (2000) further suggested that natural selection may have shaped cognitive and perceptual errors in a way that improves adaptation by minimizing the overall cost of errors. This can result in a distorted perception of reality, but such distortions may be beneficial for survival. For example, EMT posits that in environments where snakes are rare (e.g., nine sticks for every one snake), it is more adaptive to mistake a stick for a snake than to overlook an actual snake. The cost of a false positive (fleeing from a stick) is much lower than the cost of a false negative (facing a potentially fatal snake) (Haselton and Nettle 2006). These findings suggest that evolution has shaped humans to make adaptive errors that optimize survival in specific environments.

Because its initial formulation, the error management framework has been supported by various empirical studies. For instance, in sexual selection, men have been shown to overestimate women's sexual interest because the cost of missing a potential reproductive opportunity outweighs the cost of mistakenly perceiving sexual interest (Henningsen and Henningsen 2010; Koenig, Kirkpatrick, and Ketelaar 2007). Similarly, individuals are more likely to perceive out‐group members as hostile or aggressive (Brewer 1979; Quillian and Pager 2001), reflecting a bias where the cost of missing potential hostility from an out‐group member is greater than the cost of erroneously perceiving aggression. Additionally, people require minimal evidence of illness or contamination to avoid someone, whereas much more substantial evidence is needed to conclude that an individual poses no threat of contagion (Kurzban and Leary 2001; Park, Faulkner, and Schaller 2003).

The relevance of EMT to EM and EC can be understood in terms of the costs of trusting untrustworthy information versus not trusting trustworthy information. In unpredictable, hostile environments, EM may be favoured because the cost of trusting harmful or false information can be high. However, in cooperative social groups where mutual trust is essential to survival, EC may be adaptive, though context dependent. This asymmetry in error costs is influenced by the individual's social environment (Green and Phillips 2004; Raihani and Bell 2019).

Trusting untrustworthy information can lead to exploitation, reputational damage, resource loss, physical harm and even mortality (Haselton and Nettle 2006). Therefore, the costs of credulity in the face of nontrustworthy information are often greater than the costs of scepticism towards trustworthy information. However, not trusting trustworthy information carries its own risks, such as missed opportunities for learning, collaboration and social connection (Clutton‐Brock and Parker 1995; Feinberg, Willer, and Schultz 2014; Raihani, Thornton, and Bshary 2012; Robertson et al. 2014). Thus, the context‐dependent nature of these errors suggests that although EC may be adaptive in some settings, it is less beneficial in others (Haselton and Nettle 2006).

I argue that these seemingly extreme dynamics of trust are evolutionary adaptations that all humans may experience to varying degrees. In hostile environments, EM protects individuals by minimizing the harm caused by trusting untrustworthy information, as the cost of missing trustworthy information is generally lower than the cost of believing false information. In the former case, missed opportunities may occur, whereas in the latter, the consequences could be fatal. However, cooperation is also essential for survival. In some contexts, failing to trust reliable information and thus refusing to cooperate can be more dangerous than trusting untrustworthy information. As a result, the consequences of trusting false information are sometimes non‐fatal, with deception sometimes resulting in lesser harm.

However, it is reasonable to conclude that in our ancestral environment, the cost of trusting untrustworthy information was greater than the cost of failing to trust reliable information. This notion is consistent with the view that epistemic vigilance is a default human position, as evidenced by neurobiological (Bartz et al. 2011) and developmental studies (Brosseau‐Liard, Cassels, and Birch 2014; Koenig and Harris 2005).

In the context of epistemic trust, individuals who experience early adversity may exhibit heightened caution in accepting information, even in benign circumstances (false‐negative error). This caution arises from the greater potential harm of failing to detect untrustworthy individuals, given their past exposure to hostile environments. Conversely, in certain contexts, the risk of missing potential support may outweigh scepticism, leading these individuals to trust communicated information in order to survive (false‐positive error).

Early experiences of abuse and deprivation can increase sensitivity to threats, which may be adaptive in hostile environments but maladaptive in benign ones. This heightened sensitivity may cause individuals to perceive others as hostile, contributing to mistrust of communicated knowledge. Such mistrust can foster rigidity, a hallmark of psychopathology, particularly in BPD (Fonagy, Luyten, and Allison 2015). Conversely, the need for support in hostile environments may result in overly trusting behaviour, leaving individuals vulnerable to exploitation and deception. Over‐reliance on untrustworthy information may lead to identity diffusion, a core feature of BPD (Kernberg 1986), characterized by an unstable and unintegrated view of self and others.

I propose that EM and EC may serve adaptive functions in an evolutionary context, influenced by environmental factors. Consequently, humans possess the capacity for both EM and credulity. Individual variability in these capacities may be linked to the perceived costs associated with trusting or mistrusting information, which I suggest is shaped by early experiences.

Specifically, contexts that evoke a conflict‐based hostile environment, reminiscent of our ancestral past where conflicts with out‐groups were prevalent, are likely to heighten tendencies towards EM. Experiences of childhood maltreatment can significantly contribute to the perception that information is unreliable, reflecting our evolutionary heritage. Conversely, such experiences may also foster a mindset of necessity for cooperation to survive, leading to an excessive trust in communicated information.

Therefore, the variation in EM and EC may be closely related to the similarities between contemporary contexts and ancestral environments that influenced cooperation and conflict dynamics.

## 7 Discussion

In this paper, I aimed to integrate EMT into the understanding of EM and EC in individuals with BPD. Rather than viewing these trust biases as mere pathological or irrational judgment errors, I propose that they are shaped by evolutionary pressures that yield adaptive outcomes in terms of survival.

From an evolutionary perspective, the ability to navigate complex social landscapes is crucial for survival. EMT suggests that individuals develop cognitive biases based on the relative costs of making false positive errors (trusting when one should not) versus false negative errors (not trusting when one should). These biases are adaptive mechanisms for avoiding social threats like deception, particularly in environments where such errors could have serious survival implications.

In a world where deception can pose significant risks, a heightened sense of mistrust serves as a protective mechanism, alerting individuals to potential threats. This is particularly relevant for individuals with BPD, who may have histories marked by trauma and deception. Consequently, their experiences could sensitize them to cues of mistrust, leading to a defensive posture that, although adaptive in certain contexts, may hinder their ability to engage in healthy relationships.

Conversely, EC may emerge as an adaptive response in social environments where forming alliances is critical for survival. In such contexts, placing trust in others—even when there is some risk of deception—might have offered net survival advantages, especially in harsh environments where social support could be a matter of life or death. For individuals with BPD, this over‐reliance on trust may be driven by a deep‐seated need for social connection and care, reflecting a belief that the potential rewards of trust outweigh the risks, even when deception seems possible.

## 8 Implications

### 8.1 Research Implications for Future Studies

Future research should focus on the empirical validation of EMT in individuals with BPD, as it offers a framework for understanding trust biases (overmistrust or overtrust) as adaptive responses to perceived social threats. Studies could specifically examine whether BPD patients systematically exhibit false positive trust errors (overmistrust) or false negative trust errors (overtrust), using SDT to assess these patterns and validate EMT's application in the context of BPD.

Another promising area of research would be exploring how threatening versus non‐threatening cues impact epistemic mistrust and epistemic credulity in BPD patients. Signal detection analysis could help determine the effects of these cues on response bias, and experimental designs incorporating evolutionary thinking could test how different environments (benign vs. hostile) influence disruptions in epistemic trust. Manipulations involving cooperation and competition in these designs could further explore how social dynamics affect trust calibration in BPD.

Further, the signal detection framework could be employed to compare the effectiveness of different ostensive cues (e.g., eye gaze, direct address, and reflecting personal narratives) in reducing EM or EC. This research could inform the development of specific cues for psychotherapy, guiding therapists in using the most effective strategies to recalibrate trust responses in BPD patients.

Finally, studies could compare response biases between individuals with BPD and control groups to better understand how these biases relate to the perceived cost of trust errors, as predicted by EMT. This research would deepen our understanding of how BPD patients make trust‐related decisions based on perceived risks and could lead to more precise therapeutic interventions.

### 8.2 Clinical Implications

The clinical implications of this evolutionary theoretical perspective offer an alternative understanding, grounded in evolutionary science, of why current clinical practices are effective. Rather than calling for new or significantly reformed interventions, this perspective underscores how existing, evidence‐based approaches for BPD, such as mentalization‐based therapy (Luyten, Campbell, Allison, and Fonagy 2020), may already foster changes in EM and EC.

Understanding these trust biases through the lens of evolutionary psychology may inform intervention strategies. Historically, individuals with BPD have often been labelled as difficult to treat (Fonagy, Luyten, and Allison 2015). However, recognizing the adaptive roots of EM and EC, as illuminated by EMT, provides a more nuanced understanding of their experiences. This perspective underscores that the capacity for both EM and EC is an evolutionary adaptation, present in all individuals as a crucial mechanism for navigating complex social environments. By reframing these cognitive patterns as adaptations rather than deficiencies, we can work towards diminishing the stigma associated with BPD—one of the most stigmatized mental health conditions (Deans and Meocevic 2006). Such a shift in perception not only fosters empathy and understanding but also paves the way for more effective therapeutic approaches tailored to the unique relational challenges faced by individuals with BPD.

Moreover, as EMT posits, individuals with BPD may exhibit systematic cognitive biases that attribute a high perceived cost to benign environments. For example, they may tend to mistrust their partner's communication because they perceive a greater cost in being wronged, even when the actual threat of deception is low. However, the long‐term costs of this persistent mistrust, such as emotional isolation or relationship strain, may not always be evident to them. In this context, interventions that highlight the hidden costs of both types of errors—false positives (trusting untrustworthy people) and false negatives (mistrusting trustworthy people)—may be beneficial. Specifically, emphasizing the costs of mistrusting trustworthy sources, rather than focusing solely on the risks of misplaced trust, could help individuals with BPD develop a more balanced understanding of social risks.

Gradual exposure to low‐risk trust scenarios could be particularly helpful in this recalibration. Given that individuals with BPD often perceive social interactions as high‐risk, encouraging them to engage in low‐stakes trust exercises may help them recalibrate their trust mechanisms in safer settings. For instance, therapists might start with small trust‐building tasks, such as sharing something personal with a therapist or a close friend and reflecting on the outcome. Over time, this can progress to more complex social interactions, allowing patients to test their trust biases and adjust their responses to fit the modern, less threatening environment.

In addition to individual work, group therapy settings offer an evolutionary‐based avenue for recalibrating trust responses. Human ancestors relied heavily on social groups to survive, and these group dynamics would have provided an essential context for individuals to test and adapt their trust biases based on real‐time feedback. Group settings provide a similar opportunity for individuals to observe trust behaviours in others, receive feedback from peers and recalibrate their own trust responses in a relatively low‐risk social environment.

The arousal generated by their threat detection processes can also lead individuals with BPD to transition from controlled mentalizing—which involves reflective, conscious and effortful engagement—to automatic mentalizing, characterized by reflexive, subconscious processing that requires little to no effort (Luyten, Campbell, Allison, and Fonagy 2020). As a result, individuals may struggle to understand their own actions and those of others in terms of underlying mental states. This loss of controlled mentalizing, exacerbated by heightened arousal, hinders their capacity to form complex mental representations.

When automatic mentalizing takes precedence, their mental model for understanding themselves and others becomes overly simplistic, lacking the nuance necessary for effective social interaction. Consequently, the perceived cost of trusting seemingly untrustworthy information often outweighs the risks associated with mistrusting trustworthy environments. This belief reinforces the notion that the cost of trusting others is significantly higher than that of mistrust, complicating their understanding of themselves and those around them.

Thus, implementing mentalizing‐based techniques that aim to elevate the threshold from automatic to controlled mentalizing could help individuals with BPD reframe their evolutionary heritage, facilitating better social understanding and interaction shaped by their early environments.

## 9 Conclusion

In conclusion, integrating EMT into our understanding of EM and EC in individuals with BPD reframes these cognitive biases as adaptive responses shaped by evolutionary pressures. This perspective shifts the narrative from stigma to empathy, recognizing that heightened mistrust and credulity are strategies for navigating complex social environments, often rooted in early relational experiences. By developing evolutionary‐informed interventions that address these trust patterns, we can empower individuals with BPD to cultivate healthier relational dynamics.

## Conflicts of Interest

The author declares no conflicts of interest.
